# Safety of Simultaneous Robot-Assisted Resection of Colorectal Malignancy and Synchronous Liver Metastases

**DOI:** 10.3390/jcm15062424

**Published:** 2026-03-22

**Authors:** Miha Petrič, Boštjan Plešnik, Jurij Aleš Košir, Blaž Trotovšek, Jan Grosek

**Affiliations:** 1 Department of Abdominal Surgery, University Medical Center Ljubljana, Zaloska 7, 1000 Ljubljana, Slovenia; bostjan.plesnik@kclj.si (B.P.); jurij.ales.kosir@kclj.si (J.A.K.); blaz.trotovsek@kclj.si (B.T.); jan.grosek@kclj.si (J.G.); 2 Faculty of Medicine, University of Ljubljana, Korytkova 2, 1000 Ljubljana, Slovenia

**Keywords:** simultaneous resection, colorectal malignancy, liver metastases, minimally invasive surgery

## Abstract

**Introduction:** Simultaneous resection of primary colorectal malignancies and liver metastases resulted in outcomes comparable to those achieved through a two-stage procedure, while offering the advantage of a single surgical intervention. The role of the robotic approach remains underexplored because of the lack of comprehensive evidence. The objective of our study was to examine the safety of the robotic surgical platform, assess its short-term outcomes, and compare them with those of open procedures. **Methods and Material**: We retrospectively analyzed data from an initial small series of eight consecutive patients treated at the UMC Ljubljana between March 2023 and December 2025. These patients underwent robot-assisted simultaneous resection of colorectal malignancies and liver metastases. Their outcomes were compared with those of an open cohort of eight patients. **Results**: The median operative time was 334 min (range, 193–415 min). No conversions or transfusions were required. Three patients experienced severe complications, accounting for 37.5% of the cohort. The median duration of hospitalization was 9.5 days. The median number of lymph nodes retrieved was 22. Complete (R0) resection of the primary tumor was achieved in all cases (100%), whereas resection of the liver was achieved in 87.5% of the cases. Importantly, there were no instances of re-hospitalization within 30 days or mortality within 90 days. **Conclusions**: Although the rate of severe complications is relatively high, the robotic surgical platform allows for the safe simultaneous resection of colorectal malignancies and liver metastases, achieving short-term outcomes comparable to those of open surgery.

## 1. Introduction

Colorectal cancer ranks as the third most prevalent cancer globally [1], with the liver being the most common site for systemic metastasis [2]. Currently, a multimodal approach is recommended for patients with metastatic colorectal cancer to ensure a personalized treatment strategy that optimizes long-term outcomes. The primary goal of management is the complete removal of all cancers, which is linked to extended survival rates [3]. In a select group of patients, simultaneous surgical removal of colorectal cancer and resectable synchronous liver metastases can be safely performed, offering long-term oncological outcomes comparable to those achieved with staged surgical interventions [4].

While the laparoscopic approach has shown outcomes that are not inferior to the open approach, with certain advantages associated with minimally invasive techniques [5], the role of robotic modalities remains unestablished. The limited accessibility due to the higher cost of robotic platforms, along with the widespread adoption of laparoscopic techniques, is a key factor contributing to the reduced number of patients. However, the safety and suitability of robotic platforms are well established in colon [6] and rectal [7] surgeries. Robot-assisted liver procedures yield outcomes comparable to open surgery [8] and, in some cases, demonstrate superior outcomes compared to laparoscopic techniques [9]. The current evidence on the role of a robotic approach in combining colorectal and liver metastases resections remains inconclusive. Most findings come from small case series, leaving questions about patient selection, perioperative outcomes, and long-term results unresolved. A recently published systematic review and meta-analysis, which compiled a larger number of robot-assisted procedures, suggests that this approach may be feasible and safe, as well as advantageous in terms of avoiding conversion, managing complexity, and enhancing perioperative recovery [10].

The objective of our study was to assess the safety of the robotic platform compared to the open approach, with a focus on outcomes. The primary endpoints were the achievement of R0 resection and the incidence of severe postoperative complications. The secondary objectives included the incidence of 90-day mortality and short-term oncological outcomes.

## 2. Materials and Methods

A retrospective analysis was performed on data from eight consecutive adult patients who underwent robot-assisted simultaneous resection of colorectal malignancies and synchronous liver metastases between March 2023 and December 2025 at the Department of Abdominal Surgery, University Medical Center, Ljubljana. To evaluate and compare the outcomes of robot-assisted simultaneous procedures, data were collected from eight consecutive patients who underwent open simultaneous resection of colorectal malignancies and synchronous liver metastases between April 2019 and December 2025. The inclusion criteria were patients presenting with concurrent colorectal malignancy and synchronous liver metastases, as determined during a multidisciplinary meeting for simultaneous resection. Although a robotic approach has been preferred in recent years (from 2023), limited access to robotic surgical platforms necessitated the use of open or laparoscopic approaches. In cases involving a multimodal approach to liver metastases, an open approach was considered to facilitate intraoperative microwave ablation of liver metastases.

### 2.1. Patient Data Collection and Perioperative Outcome Definitions

Data were collected on the patients’ general characteristics, including age, sex, body mass index (BMI), location of the primary malignancy, site of liver metastases, number of liver lesions, American Society of Anesthesiology (ASA) score [11], and preoperative or postoperative systemic therapy. Intraoperative parameters, such as the duration of the surgical procedure, blood loss, conversion, resection of the colon or rectum, and formation of diverting ileostomy, were recorded. Liver resection was classified according to the removed liver segments based on the Couinaud classification [12] and Brisbane terminology [13]. When indicated or determined in a multidisciplinary meeting, liver metastasis microwave ablation was performed by an experienced interventional radiologist using intraoperative ultrasound guidance. Postoperative parameters included the length of hospitalization, 30-day readmission rate, and 90-day mortality rate. Postoperative complications were classified according to the Clavien–Dindo classification [14], with severe complications defined as those with a Clavien–Dindo grade of ≥3. Anastomotic leak (AL) was defined by clinical signs and imaging or intraoperative findings and classified according to the International Study Group of Rectal Cancer [15]. Postoperative liver failure [16], bile leakage [17], and hemorrhage [18] were defined using the International Study Group of Liver Surgery (ISGLS) grading system. Additionally, data on post-surgery oncological outcomes were collected, including primary tumor histology, the number of retrieved and positive lymph nodes, number and size of liver metastases, and radicality of resection (R0/R1) for primary and secondary tumors.

### 2.2. Standard Operative Approach

All procedures were conducted by a select group of experienced surgeons proficient in both open liver and colorectal surgeries, trained in robotic techniques. The da Vinci Xi robotic platform (Intuitive Surgical, Sunnyvale, CA, USA) was employed for robot-assisted hepatic and colorectal procedures. In each case, liver resection was prioritized as the initial procedure, followed by colon or rectal resection.

One day prior to surgery, all patients received a dose of indocyanine green (ICG) to enhance intraoperative liver tumor visualization, except in cases where iodine allergies contraindicated its use. The patient underwent endotracheal intubation, and a central venous line was established for fluid resuscitation and monitoring of central venous pressure. The patient was positioned in the French position, with pressure points added to prevent pressure-induced injuries to the skin and tissues. A urinary catheter was also inserted.

During the liver resection phase, the patient was positioned in a 15° anti-Trendelenburg position with a 5° tilt to the left or right, contingent upon the location of the target lesion. A pneumoperitoneum of 12 mmHg was established through a periumbilical incision. The initial steps involved adhesiolysis and intraoperative ultrasound. A liver-first resection was performed to mitigate the potential impact of the Pringle maneuver on the colonic anastomosis. When feasible, an extrahepatic or intrahepatic Glissonean pedicle approach was employed for inflow control, and transection was executed using the Pringle maneuver with the crush clamp technique, utilizing Maryland bipolar forceps and scissors. Smaller structures were divided using Maryland bipolar forceps, whereas larger structures were managed with Hem-o-lok (Teleflex, Wayne, USA) clips or ligatures. Following resection, the liver resection surface was treated with Glubrane (GEM srl, Viareggio, Italy). Drains were routinely placed along the resection line. The specimens were extracted through a Pfannenstiel incision using an Endobag (Medtronic, Minneapolis, USA). After completing the liver resection, the colorectal team redocked the robotic arms and adjusted the operating table according to the planned procedure. We typically approached colorectal resections via a medial approach, clipping the major vessels with Hem-o-lok clips and dividing them with scissors or a robotic Vessel sealer device (Intuitive Surgical, Sunnyvale, CA, USA). Once the bowel was fully mobilized, we transected the relevant mesentery with the vessel sealer. Bowel transection was performed using a robotic stapler. For right-sided colonic resections, we conducted an intracorporeal side-to-side anastomosis with the robotic stapler and closed the stapler entrance using a barbed 4-0 suture in two layers. In the case of left-sided colorectal resections, we typically performed a double-stapled circular anastomosis. We inserted the head of the circular stapler extracorporeally into the proximal bowel stump and connected it to the stapler inserted transanally to create the anastomosis. We always tested the anastomosis to ensure there was no tension and confirmed satisfactory perfusion using fluorescence angiography. Additionally, rectal anastomoses were tested for air leakage.

The postoperative course adhered to enhanced recovery protocols, including early mobilization, oral feeding, and analgesic administration. Potential complications were managed according to established principles.

### 2.3. Statistical Analysis

Despite the limited number of patients, a statistical analysis was conducted with an understanding of its restricted predictive or conclusive value. Descriptive statistics, including frequencies, percentages, means/medians, ranges, and standard deviations, were employed for the purposes of description and summary. The Fisher exact test was utilized to assess the presence of a significant association between two categorical variables. Given that the data did not follow a normal distribution, patient characteristics were compared between groups using the Mann–Whitney U test. A *p*-value of ≤0.05 was considered indicative of statistical significance.

## 3. Results

Over 32 months, we conducted eight robot-assisted simultaneous resections in patients diagnosed with colorectal malignancy accompanied by synchronous liver metastases. General patient data are presented in Table 1.

No statistically significant differences were observed between the robot-assisted and open procedures with respect to the sex, age, and ASA score of the patients. However, patients in the robot-assisted cohort exhibited significantly higher values (*p* = 0.02) than those in the open group.

In the group utilizing robot-assisted procedures, 62.5% of the patients (*n* = 5) were diagnosed due to symptoms such as bleeding, obstruction, or inflammatory signs resembling diverticulitis. Two patients were diagnosed through Slovenia’s national screening initiative, the SVIT program. Additionally, two patients with stenosing rectal carcinoma underwent laparoscopic diverting stoma procedures, specifically bipolar ileostomy and bipolar transverse colostomy, before initiating preoperative systemic therapy in conjunction with radiotherapy.

**Figure 1:**
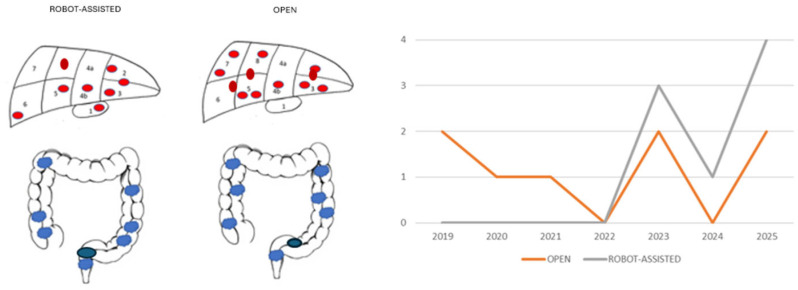
Detailed information on the primary and secondary disease burdens. Red—liver metastases; Blue—primary colorectal malignancy; *y*-axis—number of cases; *x*-axis—year.

Figure 1 provides detailed information on the primary and secondary disease burdens.

All patients in the robot-assisted procedure cohort exhibited a single metastatic lesion. In contrast, within the open surgery group, two patients presented with multiple metastatic lesions (three and four lesions, respectively).

### 3.1. Intraoperative Details

In the robot-assisted cohort, the median operative duration was notably longer compared to the open procedure cohort (334 min (93–415) vs. 146 min (120–308); *p* = 0.005). In all eight patients, both primary and secondary malignancies were removed.

Furthermore, in one patient who underwent the open approach, additional excision of the left ureter and uterus with adnexectomy was necessitated due to direct tumor invasion. In the open approach, resection of liver metastases was combined with microwave ablation in patients with multiple metastases (segments 2, 3, 5/8, and 5/6) to effectively address deep-seated metastases in segments 5/8 and 5/6. Notably, there was no conversion in the robot-assisted group.

### 3.2. Postoperative Outcomes

The details of the postoperative outcomes are listed in Table 2. Although the incidence of both overall complications (62.5% vs. 37.5%) and severe complications (37.5% vs. 12.5%) was higher in the robot-assisted group, this difference did not reach statistical significance. Furthermore, no differences were observed in transfusion rates, bile leaks, anastomotic leaks, intra-abdominal collections, or median hospital stays. The authors acknowledge that the statistical comparison is of limited value because of the small sample size and should be interpreted with caution.

In the robot-assisted group, three patients experienced severe postoperative complications, including: One patient developed a Grade C anastomotic leak, necessitating surgical intervention via the Hartmann procedure. Another patient experienced a grade C bile leak, which was initially managed with percutaneous drainage, followed by endoscopic retrograde cholangiopancreatography (ERCP). The third patient developed a suspected infected collection at the liver resection margin, and percutaneous drainage revealed sterile haemorrhagic fluid. In the open approach group, one patient developed a Grade C anastomotic leak, which also resulted in a Hartmann procedure. Intraoperatively, a biloma was identified at the site of microwave ablation (MWA) with an associated bile leak, which was treated with sutures. ERCP was performed to address the persistent bile leakage After one week, the patient developed an infection at the site of the second MWA treatment, which was managed with percutaneous drainage. Notably, there were no instances of re-hospitalization within 30 days or mortality within 90 days in either group.

### 3.3. Short-Term Oncological Outcomes

All patients in the robotic group underwent radical resection of the primary colorectal malignancy. In seven patients, liver metastases were radically resected; however, in one patient, radical resection could not be assessed due to thermocautery injury to the specimen. In comparison, seven patients in the open group underwent radical resection of both primary and secondary malignancies. The detailed short-term oncological outcomes are presented in Table 3.

Fisher’s exact test indicated no statistically significant differences in achieving R0 resection of primary colorectal malignancy and liver metastases, regardless of whether a robot-assisted or open approach was used. The Mann–Whitney U test revealed no significant differences in primary tumor size, liver metastases, or the number of retrieved and positive lymph nodes between the robot-assisted and open surgery groups. The authors acknowledge that the statistical comparison is of limited value because of the small sample size and should be interpreted with caution.

## 4. Discussion

This study demonstrated that a robot-assisted approach can be safely employed for simultaneous resection of colorectal malignancies and synchronous liver metastases. This method yields outcomes comparable to those of the open approach when executed by highly trained surgeons in a carefully selected patient cohort.

Minimally invasive approaches have certain advantages over open surgery, including reduced pain, faster recovery, and shorter hospital stays [19]. The robot-assisted approach in colorectal surgery is associated with benefits over both laparoscopic and open surgery [20,21], particularly in confined spaces such as the lower pelvis [22]. Minimally invasive approach for colorectal liver metastases (CRLMs) resection demonstrates comparable oncologic outcomes to open techniques, with a shorter length of stay (LOS), reduced blood loss, and lower complication rate [23]. Minimally invasive liver resection for colorectal liver metastases (CRLMs) yields long-term outcomes comparable to those of open surgical techniques [24]. Evidence from the literature suggests that robot-assisted liver surgery results in superior outcomes compared to the laparoscopic approach, particularly in the treatment of liver metastases located in the posterior regions [25]. In high-volume centers, the robot-assisted approach can be employed safely and feasibly in complex hepatectomy, achieving textbook outcomes [26]. Conversely, the laparoscopic approach is associated with outcomes comparable to the robotic method, while being shorter in duration and lower in cost [27]. One of the principal challenges in the advancement of robot-assisted surgery is the substantial initial cost associated with the robotic platform and hospitalization [28]. An economic evaluation indicated that in a high-volume center, robot-assisted procedures can incur similar per-procedure costs as laparoscopic procedures when capital investment is excluded [29]. These factors limit accessibility in resource-constrained countries, although price reductions may occur with the development of new, more affordable robotic platforms.

We experienced significantly longer operative times (334 (193–415) vs. 146 (120–308) min; *p* = 0.005) than the open approach group. This finding aligns with published data, where operative times ranged from 320 min [30] to 452 min [31]. Our findings are corroborated by the results of a recent meta-analysis conducted by Trindade et al. [32]., which examined a sample of 1722 patients. Of these, 210 patients (12.2%) underwent a robotic approach. This approach was associated with shorter hospital stays and reduced blood loss, while yielding comparable outcomes in terms of biliary and anastomotic leaks [32]. The robotic approach was associated with longer operative times [32]. Median operative times are challenging to interpret or compare because of the heterogeneous nature of procedures, both in primary tumor management and types of liver resections. A prolonged operative duration is associated with severe postoperative complications, including venous thromboembolism [33] and cardiopulmonary effects [34]. However, none of these complications were observed in our patient cohort. It is anticipated that, with the accumulation of knowledge and achievement of the learning curve, these procedures will gradually become shorter.

Although the incidence of overall (62.5% vs. 37.5%; *p* = 0.61) and severe (37.5% vs. 12.5%; *p* = 0.56) complications was higher in the robot-assisted group, this difference did not achieve statistical significance in our study. A meta-analysis by McGuirk et al. [35] reported a 28.6% incidence of postoperative complications, although these were not categorized according to the Clavien–Dindo classification. Machairas [36] demonstrated overall and major morbidity rates of 38% and 7%, respectively, in a meta-analysis of 29 patients undergoing simultaneous resection of colorectal malignancy and synchronous liver metastases. We acknowledge the higher incidence compared to published series, but this is likely attributable to the relatively small sample size and the prospective collection of data and complications. We found no difference in the incidence of anastomotic leak or bile leak between the robot-assisted and open approaches. These findings are supported by a recently published meta-analysis by Trindade et al. [32] Conversely, our robotic cohort was associated with a longer median hospital stay compared to the open approach, which contradicts most published data. This can be explained by the small sample size. Patients who developed C-D > 3 complications experienced prolonged hospitalization (13, 15, and 17 days), whereas the median hospitalization time for those with no postoperative complications was significantly shorter (5.5 days). One possible reason for the higher, yet not statistically significant, incidence of postoperative complications is the statistically significant higher BMI observed in the robot-assisted cohort than in the open approach. An additional factor to consider when comparing the initial eight cases performed using the robotic approach to the well-established experience with the open approach at our center is the influence of the learning curve. Despite this, no conversions were observed in our cohort, and there were no instances of 30-day re-hospitalization or 90-day mortality. The authors acknowledge that the statistical comparison is of limited value because of the small sample size and should be interpreted with caution. A larger patient series, prospective data collection, and particularly randomized controlled trials, are necessary for a comprehensive evaluation of outcomes.

The primary consideration related to patient outcomes is the oncologically adequate complete resection of both primary colorectal malignancy and synchronous liver metastases. In our study cohort, all patients underwent R0 resection of the primary tumor, with a comparable number of lymph nodes retrieved to the open group (22 vs. 25; *p* = 0.29). Radical resection of liver metastases was achieved in seven patients; however, in one patient, margin evaluation was not possible because of thermocautery tissue damage. The authors acknowledge that the R0 percentage (87.5%) is misleading because of the small sample size. In the “non-radical” case, a small 12 mm metastasis was excised with robotic scissors using diathermy, and macroscopically, the margins appeared to be adequate. Consequently, we switched to the crush clamp technique using low-power settings. Currently, the only randomized controlled trial compared outcomes between the robot-assisted and open approaches [37]. All patients in this trial underwent R0 resection of both the primary tumors and liver metastases, with a similar lymph node yield [37]. The randomized controlled trial revealed a significantly lower incidence of both overall (31.4% compared to 57.6%, *p* = 0.014) and severe (8.1% compared to 20%, *p* = 0.029) complications associated with the robotic approach as opposed to the open approach [37]. Similar reports were published by Rudnicki et al. in their initial series, who achieved R0 resection in all cases. The median operative time was 448 min (range: 374–576 min), and the number of harvested lymph nodes ranged from 16 to 49. Similarly, there were no cases of conversion [38]. All patients in our study cohort received postoperative systemic treatment and are currently alive. One patient with a mixed MiNEN tumor experienced an early progression.

The main limitation of this study is its retrospective design and limited sample size. Although statistical equivalence was noted between the open and robot-assisted groups, the small sample size undermines the robustness of the statistical analysis. This limited sample size, along with the heterogeneity of approaches, affects the study’s statistical power and influences comparative analyses. Additionally, the restricted sample size may lead to a higher complication rate and obscure potential benefits of the robotic approach. Another limitation is the possibility of selection bias. However, after acquiring the robotic platform, the last 10 patients were managed with eight using the robotic platform, one with a laparoscopic approach because of the unavailability of the robotic platform, and one with an open approach due to the use of intraoperative microwave ablation of liver metastases. Nonetheless, both the open and robotic approaches were performed by the same group of surgeons, who were proficient in open surgery and trained in robotic surgery.

## 5. Conclusions

Our initial experience, despite a relatively high incidence of complications, indicates that the robot-assisted approach to the simultaneous resection of colorectal malignancies and synchronous liver metastases is safe and yields outcomes comparable to those of open procedures. This highly selected group of patients should be managed in tertiary, high-volume centers with established expertise in the field. Further randomized controlled trials comparing robotic, open, and laparoscopic approaches are necessary to properly evaluate the position of the robotic surgical platform.

## Figures and Tables

**Table 1:** General patient data.

	ROB (*n* = 8)	OPEN (*n* = 8)	*p*
Male (%)	4 (50%)	4 (50%)	1.00
Median age (years, range)	62 (52–69)	71 (49–95)	0.06
Median BMI (kg/m^2^, range)	26.3 (18.9–34.8)	22.5 (18.3–32.1)	0.02
ASA score			1.00
2	6 (75%)	5 (62.5%)	
3	2 (25%)	3 (37.5%)	

ROB—robot-assisted resection; OPEN—open resection; *n*—number; BMI—body mass index; ASA—American Society of Anesthesiology score.

**Table 2:** The postoperative outcomes.

	ROB (*n* = 8)	OPEN (*n* = 8)	*p*
Overall complications	5 (62.5%)	3 (37.5%)	0.61
Severe complication (C-D > 3)	3 (37.5%)	1 (12.5%)	0.56
Transfusion rate	0	3 (37.5%)	0.20
Bile leak	1 (12.5%)	1 (12.5%)	1.00
Anastomotic leakage	1 (12.5%)	1 (12.5%)	1.00
Intraabdominal abscess/fluid collection	1 (12.5%)	1 (12.5%)	1.00
Median hospitalization (days, range)	9.5 (4–20)	7.5 (7–55)	0.31

ROB—robot-assisted resection; OPEN—open resection; *n*—number; C-D (Clavien–Dindo).

**Table 3:** Short-term oncological outcomes.

	ROB (*n* = 8)	OPEN (*n* = 8)	*p*
R0 primary malignancy (%)	8	7 (87.5%)	1.00
Size (mm)	17.5 (15–65)	30 (14–68)	0.26
N (median, range)	22 (11–35)	25 (13–89)	0.29
N poz. (median, range)	1 (0–11)	1.5 (0–58)	0.28
N of liver metastases (median, range)	1	1 (1–4)	1.00
R0 liver metastases (%)	7 (87.5%)	7 (87.5%)	1.00
Size (mm)	25 (10–47)	15 (12–50)	0.23

ROB—robot-assisted resection; OPEN—open resection; *n*/N—number.

## Data Availability

Data is available upon request.
